# Molecular and histopathological landscape of 131 meningiomas: a retrospective institutional study with insights from cIMPACT-NOW

**DOI:** 10.3389/fonc.2025.1648953

**Published:** 2025-08-29

**Authors:** Rola H. Ali, Abdulaziz Hassan, Hussain H. Jarkhi, Abdullah Alshawish, Mohamad Almanabri, Obada T. Alhalabi, Ahmad R. Alsaber, Nawal Y. Ali, Ehab Abdelnabi, Eiman M. A. Mohammed, Hiba Jama, Ammar Almarzooq, Zainab Alqallaf, Amir A. Ahmed, Shakir Bahzad, Stefan Hamelmann, Felix Sahm, Maryam Almurshed

**Affiliations:** ^1^ Department of Pathology, College of Medicine, Kuwait University, Jabriya, Kuwait; ^2^ Department of Histopathology, Al Sabah Hospital, Shuwaikh, Kuwait; ^3^ Department of Diagnostic Radiology, Jaber Alahmad Hospital, South Surra, Kuwait; ^4^ Department of Neurosurgery, Jaber Alahmad Hospital, South Surra, Kuwait; ^5^ Department of Neurosurgery, Heidelberg University Hospital, Heidelberg, Germany; ^6^ Department of Management, College of Business and Economics, American University of Kuwait, Salmiya, Kuwait; ^7^ Department of Radiology, Ibn Sina Hospital, Shuwaikh, Kuwait; ^8^ Molecular Genetics Laboratory, Kuwait Cancer Control Center, Shuwaikh, Kuwait; ^9^ Department of Neuropathology, Heidelberg University Hospital, Heidelberg, Germany

**Keywords:** meningioma, molecular sequencing, NF2, WHO grade, cIMPACT-NOW

## Abstract

**Background:**

Prognostication in meningiomas has traditionally relied on histopathological grading, which has inherent limitations, including interobserver variability, intratumoral heterogeneity, and inconsistent correlation with clinical behavior. While molecular profiling enhances diagnostic precision and risk stratification, it is not yet routinely adopted in clinical practice. To date, no molecular data on meningiomas have been published from our country. This study aims to address this gap by characterizing the molecular landscape of meningiomas at our institution, incorporating insights from recent cIMPACT-NOW updates.

**Methods:**

We retrospectively analyzed consecutive 131 meningiomas that underwent molecular sequencing at our institution between 2021 and 2023. Tumors were classified according to the latest WHO criteria. Next-generation sequencing (NGS) was performed using the Oncomine Comprehensive Assay, a targeted panel for solid tumors. Molecular findings were correlated with clinicopathological parameters.

**Results:**

The cohort included 84 females and 47 males (median age: 51 years; range: 2–79). Tumor locations included the cerebral convexity (45.8%), skull base (38.2%), posterior fossa (3.1%), and spine (5.3%), with 7.6% being multifocal. CNS WHO grade 2 tumors were most common (58%), followed by grade 1 (35%) and grade 3 (7%). *NF2* alterations (35%) were the most frequent, occurring across all grades but more prevalent in grades 2 and 3. Genotype (p = 0.004) and WHO grade (p = 0.002) were significantly associated with tumor location: *NF2* alterations predominated in convexity and spine, while TRAKLS mutations (*TRAF7*, *AKT1*, *KLF4*, *SMO*) were enriched in lower-grade skull base tumors. High-risk homozygous *CDKN2A/B* deletions were identified in one grade 3 tumor, with hemizygous deletions, unexpectedly, in three grade 2 tumors.

**Conclusion:**

This study provides regional insight into the molecular landscape of meningiomas in our population. While routine molecular profiling adds value to classification and prognostication, broader implementation may be limited by cost and panel coverage constraints.

## 1 Introduction

Meningioma is the most common primary intracranial neoplasm in adults, originating from arachnoid cap cells within the leptomeninges (1, 2). Grading of meningioma continues to rely primarily on histological criteria, as defined by the three-tier system in the current World Health Organization guidelines (WHO CNS5, 2021) (3). CNS WHO grade 1 meningiomas are generally slow-growing and associated with favorable outcomes, while CNS WHO grade 2 and 3 tumors demonstrate more aggressive behavior and increased risk of recurrence (4). However, recurrence remains a complex and nuanced challenge, as meningiomas can exhibit inconsistent behavior, even among grade 1 tumors (5, 6). This is further influenced by clinical factors, particularly the extent of surgical resection (7). Given these complexities, the incorporation of relevant molecular parameters that would reliably predict meningioma behavior has become increasingly important. The “Consortium to Inform Molecular and Practical Approaches to CNS Tumor Taxonomy-Not Official WHO” (cIMPACT-NOW) has recently clarified the role of molecular markers in meningioma grading, emphasizing their potential clinical relevance (8).

Rapid advances in genomic profiling have uncovered distinct molecular subgroups of meningiomas, which correlate with unique clinical characteristics including tumor location, histological subtype, and WHO grade (9). While *NF2* gene inactivation has long been recognized as a key driver of meningioma tumorigenesis (10, 11), more recent studies have identified non-*NF2* alterations, particularly those involving the TRAKLS genes (*TRAF7*, *AKT1*, *KLF4*, *SMO*), as well as other genetic changes not otherwise classified (12, 13). In terms of clinical behavior, a few rare molecular alterations have been linked to aggressive meningioma biology, including oncogenic variants in the *TERT* promoter region and homozygous deletions of *CDKN2A/CDKN2B* (14, 15). These are frequently observed alongside *NF2* alterations, reflecting a stepwise accumulation of genetic changes driving tumor progression (16–18). However, in the absence of these high-risk mutations, no reliable biomarkers currently exist to predict the risk of recurrence in the more prevalent lower-grade meningiomas. A growing body of literature supports the integration of genomic, epigenomic, and transcriptomic parameters into meningioma classification, offering enhanced predictive value and potential for personalized therapeutic approaches (19, 20). However, the widespread clinical implementation of such molecular tools remains constrained by cost, availability, and accessibility.

To complement histopathological evaluation and gain deeper insight into meningioma-associated molecular events, we implemented routine targeted sequencing for or all meningioma specimens at our laboratory beginning in 2021. In this study, we analyze data from 131 meningiomas diagnosed over the past three years, exploring the mutational landscape and its associations with clinicopathological parameters, with a focus on WHO grading refinements.

## 2 Materials and methods

### 2.1 Case selection

Meningioma cases diagnosed between 2021-2023 were retrospectively retrieved from the electronic records of the Department of Pathology at Sabah hospital, a major neuropathology site in Kuwait. Demographic and clinicopathological data were obtained from pathology reports and patient records.

### 2.2 Radiological and clinical data

Preoperative and postoperative magnetic resonance imaging (MRI) data were available for 105 (80%) and 118 (90%) patients, respectively. Tumor locations on MRI were categorized as follows: cerebral convexity (including falcine/parasagittal regions); skull base (including structures related to the sphenoid bone, ethmoid bone, and petroclival region in the posterior fossa); miscellaneous posterior fossa locations (e.g., cerebellopontine angle, tentorium cerebelli, cerebellar convexity); and spinal cord. Cases with ≥2 meningiomas at different locations were classified as multifocal. Other radiological parameters included tumor size, necrosis, cystic change, peri-tumoral brain edema, and parenchymal/skull invasion. Postoperative MRI was utilized to detect residual disease. Clinical follow-up data were obtained for 81 patients, and recurrence-free survival was calculated from the date of the first surgery to the first recurrence.

### 2.3 Histopathology review

Slides were re-evaluated by a neuropathologist according to the 2021 WHO Classification of CNS Tumors and the 2024 cIMPACT-NOW Update. Grade 2 was assigned based on any of the following criteria: ≥4 mitoses per 10 high-power fields (HPF), brain invasion, >50% chordoid or clear cell histology, or at least three of the following five morphological features: (i) hypercellularity, (ii) sheeting architecture, (iii) small cell change, (iv) prominent nucleoli, and (v) spontaneous necrosis. Grade 3 was assigned for tumors with ≥20 mitoses per 10 HPF, frank anaplasia, and/or molecular evidence of oncogenic *TERT* promoter variants or homozygous deletions of *CDKN2A/CDKN2B*. Immunohistochemical slides were reviewed as needed. Mitoses were manually counted in hotspots across 10 consecutive HPFs using a microscope with an objective lens providing a field area of 0.24 mm². The mitotic counts were adjusted to 1 mm², applying thresholds of ≥2.5 mitoses/mm² and ≥12.5 mitoses/mm² for grade 2 and grade 3 tumors, respectively.

### 2.4 Targeted next-generation sequencing

All sequencing and bioinformatics analyses were performed in-house at the Molecular Genetics Laboratory of the Kuwait Cancer Control Center. Formalin-fixed, paraffin-embedded (FFPE) tumor tissues were analyzed using next-generation sequencing (NGS) with the Oncomine Comprehensive Assay v3 (OCAv3; Thermo Fisher Scientific, USA) (21). This panel detects single-nucleotide variants, insertions/deletions, copy number variants, and gene fusions across 161 cancer-related genes (**Supplementary Table 1**), including meningioma-relevant genes listed in **Table 1**. DNA extraction was performed using the RecoverAll Total Nucleic Acid Isolation Kit, followed by library preparation with the Ion AmpliSeq Library Kit Plus (Thermo Fisher Scientific, USA). DNA concentration was measured using the Qubit 3.0 Fluorometer, which offers fluorometric quantification with high specificity for double-stranded DNA. Extraction from FFPE material was successful in the majority of cases; however, three specimens exhibited excessive nucleic acid degradation and failed downstream library preparation or sequencing quality control metrics, leading to their exclusion from analysis.

**Table 1:** Meningioma-relevant genes included in the NGS panels.

Initial general cancer panel		
Genes	Loci	Significance/ associations
*AKT1* (13, 23)	14q32.33	Enriched at skull base
*BAP1* (24, 25)	3p21.1	High-grade rhabdoid/papillary subtype; associated with germline defects
*CDKN2A/CDKN2B* (15, 16, 26)	9p21.3	Homozygous deletions associated with progression
*CREBBP* (27)	16p13.3	Associated with germline defects (Rubinstein-Taybi syndrome)
*NF2* (28, 29)	22q12.2	Mutated in 40%-60% of meningiomas; most common germline defect predisposing to meningioma
*PIK3CA* (30)	3q26.32	Enriched at skull base
*PIK3R1* (31)	5q13.1	–
*PTCH1* (32)	9q22.32	Associated with germline defects (Gorlin syndrome)
*PTEN* (33)	10q23.31	Associated with germline defects (Cowden syndrome)
*SMARCB1* (34)	22q11.23	Associated with germline defects
*SMO* (13, 23, 35)	7q32.1	Enriched at skull base in olfactory groove
*TERT* (14, 17)	5p15.33	Promoter mutation associated with progression

Customized brain tumor panel		
Additional genes	Loci	Significance/ associations
*KLF4* (13, 36)	9q31.2	Co-occurs with TRAF7 mutations especially in secretory subtype
*PBRM1* (37)	3p21.1	Papillary subtype
*PRKAR1A* (38)	17q24.2	–
*SMARCE1* (39, 40)	17q21.2	Clear cell subtype; associated with germline defects
*SUFU* (41)	10q24.32	Associated with germline defects
*TRAF7* (13)	16p13.3	Enriched at skull base
*VHL* (42)	3p25.3	Associated with germline defects (von Hippel-Lindau disease)
*YAP1* (43)	11q22.1	Predominantly pediatric patients

Sequencing was conducted on an Ion Torrent S5 XL platform, with data analyzed using the Ion Reporter™ Software (v5.10). Sequence reads were aligned to the human genome assembly GRCh37/hg19, achieving a minimum of 10 million total mapped reads. Variant calling was conducted with a minor allele frequency cutoff of 5% and a mean depth of ≥200x. To ensure sequencing reliability, stringent quality metrics were applied, including base quality scores (Q30 or higher), mapping quality (MQ ≥60), and uniformity of coverage (≥90%). Duplicate reads were filtered out when exceeding a 5% threshold. Variant calling further required a minimum of 20 supporting reads, an allele balance of at least 20%, and strand bias ≤10%. Annotations were obtained using databases such as ClinVar, COSMIC, and dbSNP, with pathogenicity assessed using tools like SIFT and PolyPhen-2. Subsequently, 24 mutation-negative tumors underwent further sequencing using a customized 201-gene NGS panel targeting brain tumor–relevant alterations (PANEL-NPHD2022A) performed on a NextSeq 500 instrument (Illumina) as previously described (22).

### 2.5 Statistical analysis

Descriptive statistics were used for continuous variables (mean, median, range, and standard deviation), as well as categorical variables (frequencies and graphical representations). Clinicopathological parameters were evaluated using univariate analysis: Pearson’s Chi-squared test for categorical variables and a two-sample independent t-test for continuous variables. A p-value <0.05 was considered statistically significant. The statistical analysis was conducted using JAMOVI Version 2.5.7.0.

## 3 Results

### 3.1 Clinicopathological findings

**Figure 1:**
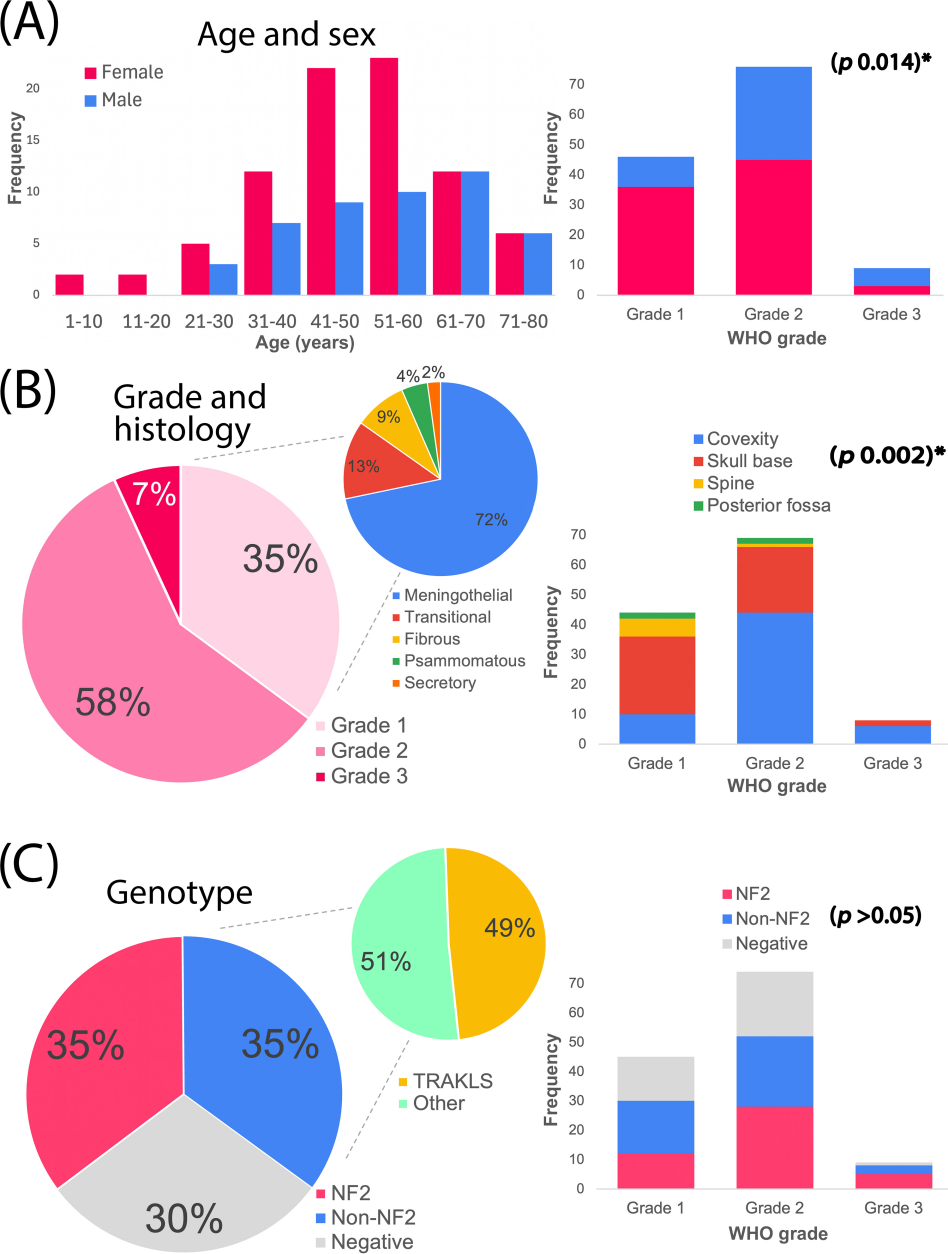
Overall characteristics of the meningioma cohort. **(A)** Age and sex distribution, alongside sex breakdown by WHO grade. **(B)** WHO histological grades and subtypes, alongside meningioma locations across grades. **(C)** Genotype groups, with genotype distribution across grades. TRAKLS= TRAF7, AKT1, KLF4, SMO alterations. *Statistically significant.

We analyzed 131 surgically resected meningiomas for genetic alterations from 2021 to 2023. Key clinicopathological features are summarized in **Table 2**. The cohort included 84 females and 47 males (female-to-male ratio ≈ 2:1), with a median age of 51 years (range 2–79) (**Figure 1A**). Three pediatric patients <18 years were identified. Most meningiomas were intracranial, with the cerebral convexity as the most common location (n=60, 45.8%), followed by skull base (n=50, 38.2%), miscellaneous posterior fossa locations (n=4, 3.1%), and spine (n=7, 5.3%). Ten (7.6%) were multifocal, including two syndromic neurofibromatosis type 2 (NF2) cases.

**Table 2:** Clinicopathological characteristics.

Characteristics	n (%)
Age	
Mean (SD)	50.7 (14.9)
Range	2.0 - 79.0
Sex	
Female	84 (64.1%)
Male	47 (35.9%)
Tumor size (cm)	
Mean (SD)	4.4 (1.9)
Range	1.3 - 12.0
Tumor location	
Cerebral convexity	60 (45.8%)
Skull base	50 (38.2%)
Miscellaneous posterior fossa	4 (3.1%)
Spine	7 (5.3%)
Multifocal	10 (7.6%)
CNS WHO grade	
Grade 1	46 (35.1%)
Grade 2	76 (58.0%)
Grade 3	9 (6.9%)
Histological subtype	
Meningothelial	33 (25.2%)
Transitional	6 (4.6%)
Fibroblastic	4 (3.1%)
Psammomatous	2 (1.5%)
Secretory	1 (0.8%)
Atypical	73 (55.7%)
Chordoid	2 (1.5%)
Clear cell	1 (0.8%)
Anaplastic +/- papillary, rhabdoid	9 (6.8%)
Surgery type	
Gross total resection	59 (74.7%)
Subtotal resection	20 (25.3%)
Not available	52
Total	131

WHO histological grades were distributed as follows: 46 (35%) grade 1, 76 (58%) grade 2, and 9 (7%) grade 3 (**Figure 1B**). Grade 1 tumors included meningothelial (n=33), transitional (n=6), fibroblastic (n=4), psammomatous (n=2), and secretory (n=1) subtypes. Grade 2 tumors (largest group) comprised atypical meningiomas (n=73), chordoid (n=2), and clear-cell (n=1) variants. Grade 3 (anaplastic) meningiomas (n=9) included 3 cases with rhabdoid/papillary features.

**Figure 2:**
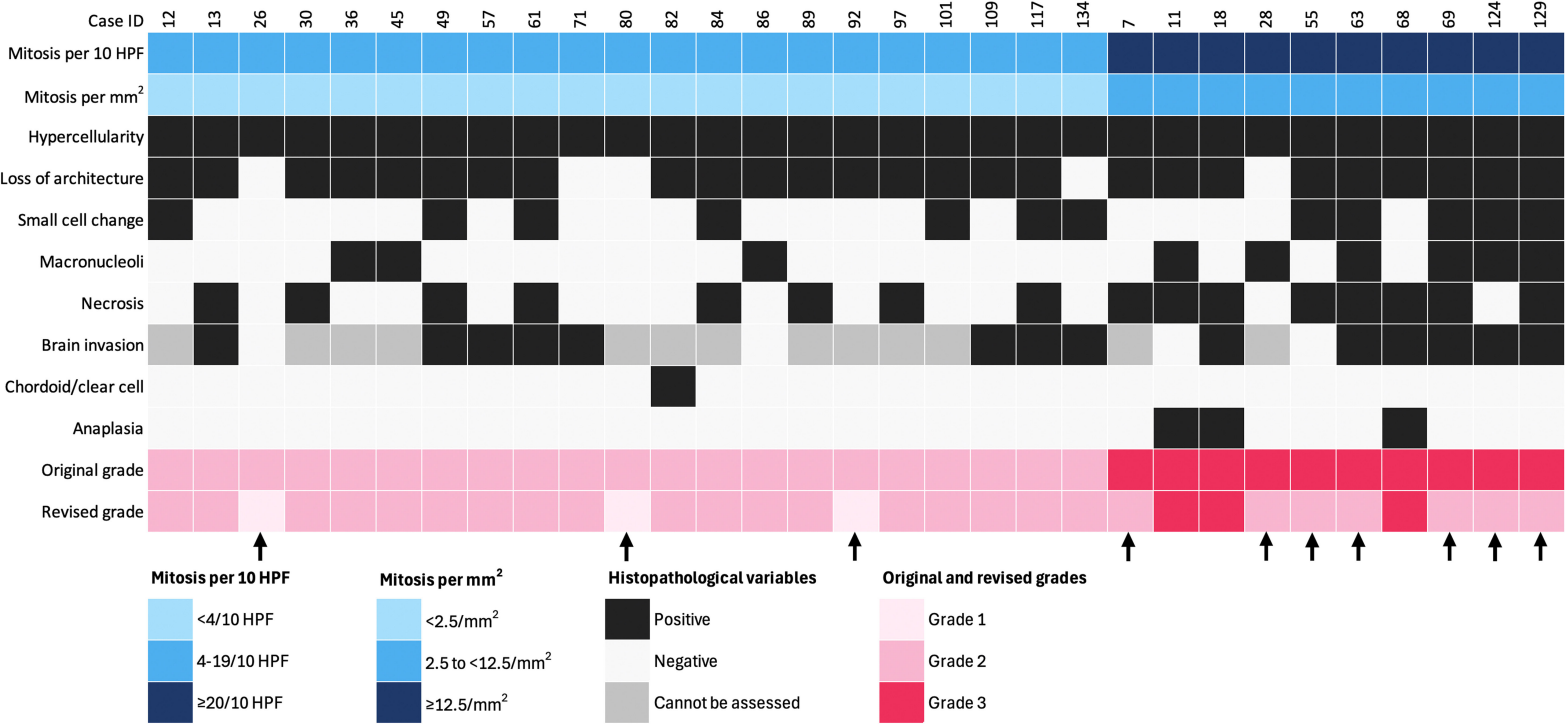
Impact of mitotic count adjustment on tumor grade in 31 cases (1 HPF = 0.24 mm^2^). Arrows indicate downgraded cases. Each column represents an individual patient, and each row corresponds to a specific variable.

Grade 2 was assigned based on morphological/brain invasion criteria in 37%, morphology alone in 26%, mitotic/morphological criteria in 19%, and all three criteria in 13% of tumors. Additionally, 4% were clear-cell or chordoid variants, and 1% exhibited isolated brain invasion (brain-invasive otherwise benign; BIOB). Grade 3 tumors were classified based on anaplasia +/- mitotic criteria (56%) or mitotic criteria alone (44%). The grades were determined following the conversion of mitotic counts from 10 high-powered fields (HPFs) to 1 mm², using mitotic cutoffs of ≥2.5/mm² and ≥12.5/mm² for grades 2 and 3, respectively. This adjustment changed the mitotic category in 31 cases, ultimately leading to the downgrading of 3 tumors to grade 1 and 7 tumors to grade 2 (**Figure 2**).

### 3.2 Mutation analysis

Of the 128 successfully sequenced tumors, 115 (90%) were newly diagnosed (*de novo*), and 13 (10%) were recurrent. Genetic alterations were detected in 90 samples (70%), including 45 tumors (35%) with *NF2* alterations, 45 (35%) with non-*NF2* alterations, and 38 (30%) with no detectable alterations (**Figure 1C**). The annotated genetic variants and their allelic frequencies are provided in **Supplementary Table 2**.

**Figure 3:**
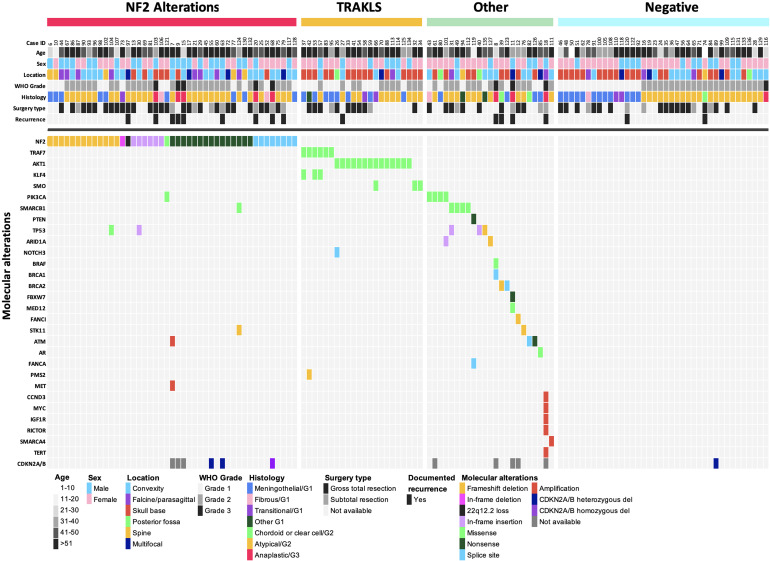
Oncoplot summary (n = 128). Each column represents an individual patient, and each row corresponds to a specific variable.

*NF2* alterations (n=45) were diverse, including frameshift deletions (n=13), in-frame deletion (n=1), nonsense mutations (n=15), frameshift insertions (n=6), splice site mutations (n=8), missense mutation (n=1), and heterozygous 22q12.2 loss (n=1) (**Figure 3**). The two syndromic NF2 tumors harbored a splice site mutation and a heterozygous 22q12.2 loss, respectively.

In the non-*NF2* subgroup (n=45), a total 26 TRAKLS mutations were identified across 22 tumors, which were mutually exclusive of *NF2* mutations. These included missense mutations in *TRAF7* (n=6), *AKT1* (n=14), *KLF4* (n=3) and *SMO* (n=3). Recurrent variants were as follows: *AKT1* p.E17K (n=12), *KLF4* p.K409Q (n=3), and *SMO* p.L412F (n=2). *TRAF7* mutations exhibited variability, often co-occurring with *KLF4* p.K409Q. Other non-*NF2* alterations were heterogenous involving *SMARCB1*, *PIK3CA*, *TP53*, and various other genes (**Supplementary Table 2**). *NF2* co-mutations were observed with *TP53* (n=2), *PIK3CA* (n=1), and *SMARCB1/STK11* (n=1).

### 3.3 Clinicopathological and genotype correlations

**Figure 4:**
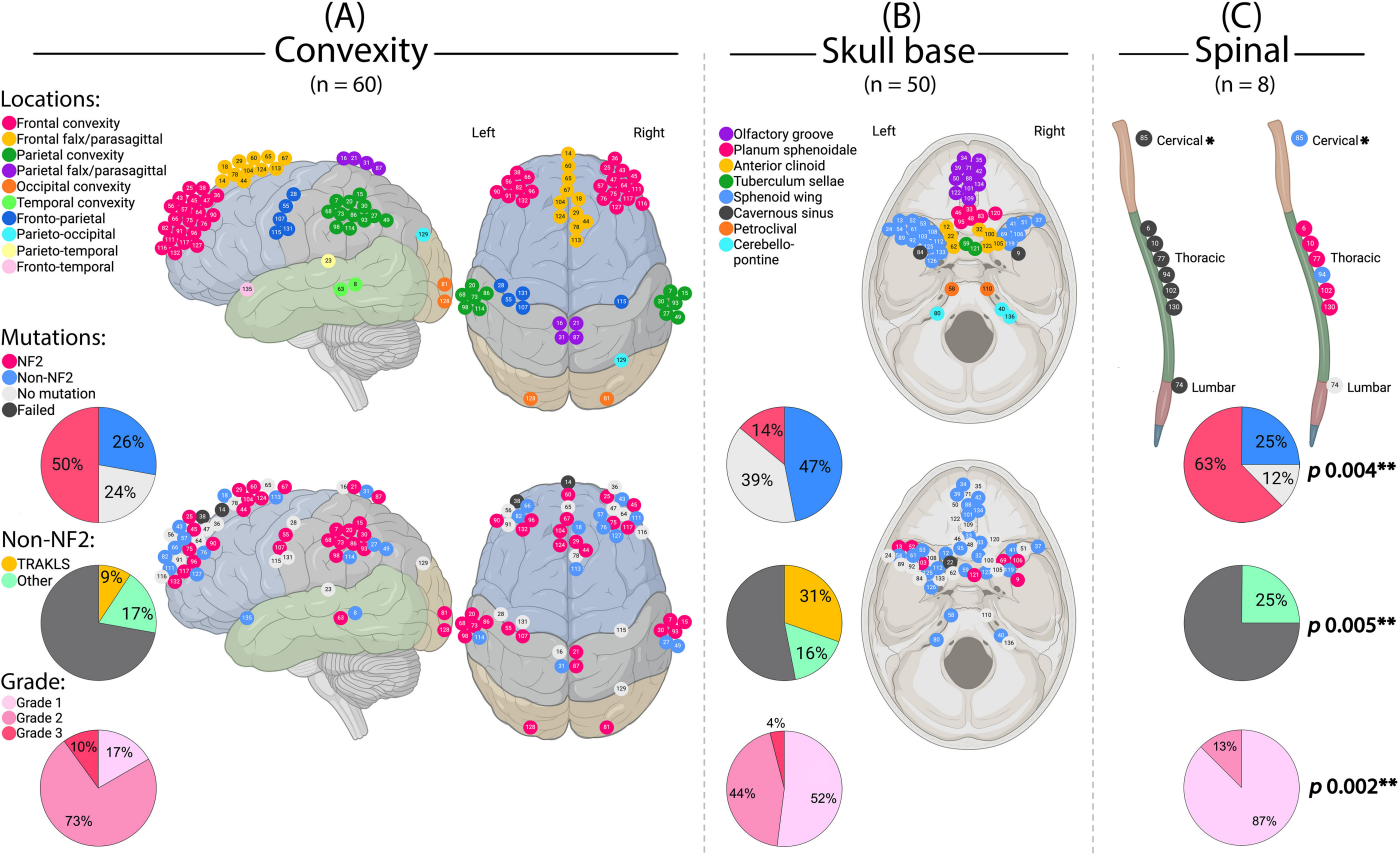
Meningioma location in relation to genotype and grade. **(A)** Cerebral convexity, sagittal and axial views. **(B)** Skull base, axial view. **(C)** Spinal cord. *This case also had another meningioma of the cavernous sinus. **Statistically significant. (Created by RHA with BioRender.com).

Genotype correlated with tumor location (p = 0.004). *NF2* alterations predominated in cerebral convexity (29/58; 50%) and spinal tumors (5/7; 71%), while TRAKLS mutations were enriched in skull base tumors (15/22; 68%) (**Figure 4**). Tumor size was significantly larger in the *NF2* subgroup (mean 4.9 cm; p = 0.013). Qualitative radiological features (e.g., necrosis, edema, peritumoral brain edema, parenchymal/skull invasion) did not differ among genotypes.

WHO grade also correlated with location (p = 0.002), with grade 1 tumors predominantly found at the skull base and higher grades at the convexity (**Figure 4**). Grade 3 tumors were more common in males (p = 0.014) and associated with necrosis (p = 0.002), cystic changes (p = 0.004), and perilesional edema (p = 0.002) on imaging.

**Figure 5:**
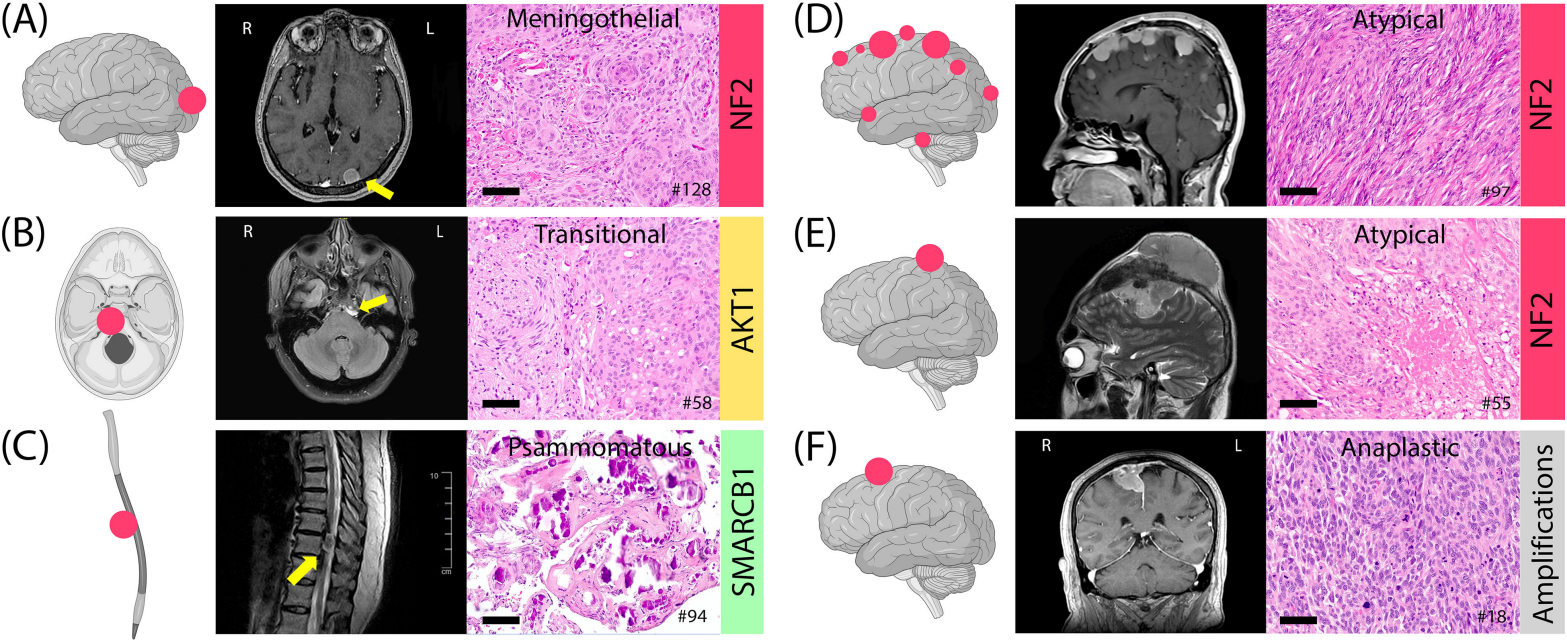
Examples of meningiomas with location, histology, MRI, and genotype. **(A)** Meningothelial meningioma, occipital convexity, grade 1. **(B)** Transitional meningioma, petroclival, grade 1. **(C)** Psammomatous meningioma, thoracic spine, grade 1. **(D)** Multifocal atypical meningiomas in NF2 syndrome with a heterozygous 22q12.2 loss, grade 2. **(E)** A 12-cm atypical meningioma, fronto-parietal convexity, with intra- and extra-axial components, grade 2. **(F)** Anaplastic meningioma with sarcoma-like histology, frontal parasagittal, grade 3. Histology images at 20x magnification; scale bars= 100µm.

*NF2* alterations were more frequent in WHO grade 2 and 3 tumors (grade 1: 26%; grade 2: 37%; grade 3: 56%) (**Figure 5**). When combined, grade 2 and 3 tumors comprised 73% of the *NF2* subgroup. In contrast, the TRAKLS subgroup included only grade 1 (50%) and grade 2 (50%) tumors, with no grade 3 cases. *CDKN2A/B* homozygous loss was found in one grade 3 *NF2*-mutated tumor, while hemizygous loss was detected in three grade 2 cases. No *TERT* promoter mutations were identified.

Brain invasion was observed in 45 of 66 specimens containing brain parenchyma (40 grade 2 tumors and 5 grade 3 tumors). No molecular correlation with invasion was identified. One *NF2*-mutated BIOB tumor showed isolated brain invasion without mitotic activity or atypical morphological criteria.

**Figure 6:**
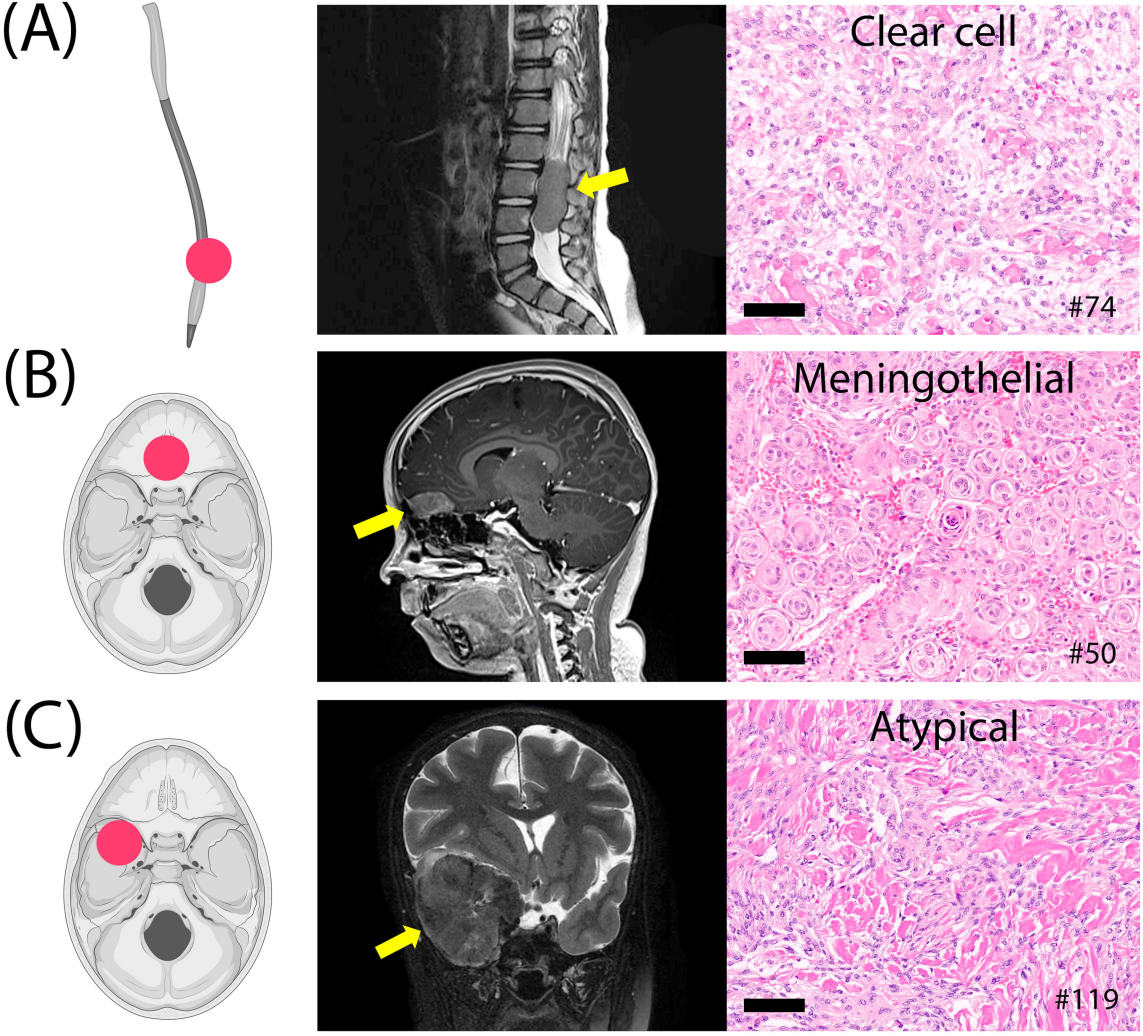
Three pediatric meningiomas. **(A)** Clear cell meningioma of the lumbar spine, grade 2. **(B)** Meningothelial meningioma of the olfactory groove, grade 1. **(C)** Atypical fibroblastic meningioma of the sphenoid wing, grade 2, post-radiation therapy. Histology images at 20x magnification; scale bars= 100µm.

Three pediatric cases were included: a spinal clear-cell meningioma (grade 2), an olfactory groove meningothelial meningioma (grade 1), and a sphenoid wing atypical meningioma (grade 2) with *PTEN* and *FANCA* pathogenic mutations (**Figure 6**). The latter case followed prior radiation therapy for medulloblastoma. The other two cases did not show detectable genetic alterations using the assay employed.

Post-operative imaging (n=118) revealed residual tumors in 37 cases (31.4%), with significantly higher rates in subtotal resection (STR; 90%) compared to gross total resection (GTR; 17%) (p < 0.001). Residual disease was most common in grade 3 tumors (67%) but did not correlate with genotype. GTR was more frequently achieved in convexity and spinal tumors.

Clinical follow-up in 81 patients (median: 2.75 years) revealed recurrences in 16 cases (20%), significantly associated with WHO grade (p < 0.001): 89% in grade 3, 9% in grade 2, and 2% in grade 1 tumors. Among the recurrent cases, recurrence-free intervals ranged from 7.6 to 149.5 months (median: 46.6 months). Due to inconsistent follow-up documentation across the broader cohort, formal recurrence-free survival (RFS) analysis could not be performed. Recurrence rates did not differ significantly between genotype groups (p = 0.245), though the TRAKLS subgroup exhibited the lowest recurrence rate (4.5%). Syndromic NF2 patients experienced multiple recurrences requiring surgical reinterventions and gamma knife therapy. Among the 16 recurrent cases, 5 had undergone GTR, including 3 cases with no visible postoperative residual disease. All recurrent cases with GTR were grade 2 or 3 tumors. Tumor location showed no association with recurrence.

## 4 Discussion

Meningioma diagnostics has traditionally relied on histological criteria as the primary means of predicting outcomes, with the incorporation of molecular parameters being a more recent development. In this study, we present our analysis of 131 molecularly sequenced meningiomas, exploring the mutational profiles of the cohort and their associations with histopathological and clinical parameters. To our knowledge, this is the first comprehensive molecular profiling study of meningiomas conducted in Kuwait and one of the very few from the Middle East. While the genomic alterations identified align with previously reported patterns, the study offers significant regional value and contributes data from an underrepresented population. Notably, the median age of our cohort was 51 years, which is younger than the global median age of 66 years reported in the WHO Classification of CNS Tumours (3); whether this reflects demographic differences remains to be explored. In the context of evolving molecular classification frameworks, documenting institutional experiences, particularly from resource-limited settings, remains important for ensuring diagnostic consistency and guiding future research.

As anticipated, *NF2* alterations represented the most frequent genetic abnormality. These were notably associated with convexity and spinal meningiomas, occurring across all grades but with enrichment in higher-grade tumors. While *NF2* alterations are not direct predictors of prognosis or strictly correlated with WHO grading, they are linked to a more aggressive biological phenotype (11, 44). The *NF2* mutational spectrum in our cohort was broad, with nonsense mutations and frameshift deletions being the most common, as described elsewhere (45). In contrast, TRAKLS alterations were more prevalent in lower-grade tumors at the skull base, in line with prior findings (46). The relatively high frequency of mutation-negative cases in our study (30%) can be attributed to the limited coverage of the NGS assay used, which did not capture several known meningioma-related alterations. Notably, a customized brain tumor–specific panel improved detection by 42% among 24 mutation-negative cases that were further tested. Histological subtype–specific mutations, such as *KLF4/TRAF7* in secretory meningiomas (47, 48) and *SMARCE1* in clear cell meningiomas (39, 40), were also not supported by our assay.

Homozygous deletions of *CDKN2A/B* and oncogenic *TERT* promoter mutations are well-established independent adverse prognostic factors and defining features of CNS WHO grade 3 meningiomas. In this study, homozygous *CDKN2A/B* loss was observed in a single grade 3 meningioma, while all histologically low-grade tumors retained intact *CDKN2A/B* and *TERT* loci. Interestingly, three grade 2 meningiomas displayed hemizygous *CDKN2A/B* loss, a finding that has been linked to poor outcomes in some studies (18, 49). Oncogenic *TERT* promoter variants, though rare, have been reported in lower-grade meningiomas (14). A recent study also identified significant discrepancies between histological grades and molecular profiles, highlighting the critical role of molecular screening in refining meningioma classification and prognostication (50). These findings reinforce the value of integrating routine molecular profiling to complement traditional histological grading and improve risk stratification. However, widespread adoption of routine meningioma genotyping in clinical practice remains constrained by cost-effectiveness concerns.

Three pediatric meningiomas were identified in this cohort: a grade 2 clear-cell meningioma in the spinal region, a grade 1 tumor in the olfactory groove with nasal extension, and a grade 2 sphenoid wing tumor that developed years after radiation therapy for medulloblastoma. Pediatric meningiomas are rare, comprising about 1% of all meningiomas, and have distinct clinicopathological and genetic features compared to adults (51). These include a lack of female preponderance (52), higher incidence of spinal and intraventricular localizations (52, 53), higher grade histopathological features (54, 55), and more aggressive histological subtypes such as clear-cell meningioma (56). While *NF2* alterations are frequent in pediatric meningiomas, with a higher frequency of underlying NF2 syndrome (54), adult-type non-*NF2* alterations are typically absent (57). Alternative alterations, such as those in *YAP1*, have been described (43, 58). DNA-methylation profiling of pediatric cases also differs from adults (57). No *NF2* alterations were found in our pediatric cases, and we could not assess *SMARCE1* mutations in the clear-cell meningioma due to limited NGS panel coverage. The post-radiation case showed mutations in *PTEN* and *FANCA*, though the significance of these remains uncertain (59).

Our cohort demonstrated a higher frequency of grade 2 meningiomas (58%) compared to grade 1 (35%), which exceeds what is typically reported in the literature (60). This discrepancy likely reflects cumulative factors beyond mitotic count thresholds or brain invasion alone. While we adopted recommended practices—such as full tissue processing and adjusting mitotic counts from 10 HPFs to mm²—we also observed a high frequency of subjective morphological features that contribute to grade 2 designation: hypercellularity in 84% (110/131), loss of architecture in 67% (88/131), necrosis in 37% (49/131), small cell change in 32% (42/131), and prominent nucleoli in 27% (36/131). Grade 2 was assigned based on combined morphological and brain invasion criteria in 37% of cases, morphology alone in 26%, morphology plus mitotic counts in 19%, and all three criteria in 13%. These findings may suggest a tendency toward overinterpretation of soft histological features which are inherently subjective. Nonetheless, we view this as a valuable institutional audit—offering insight into how WHO grading criteria are implemented in real-world diagnostic environments. While large-scale studies establish reference standards, single-institution experiences like ours remain essential for highlighting diagnostic variability, informing local quality assurance, and encouraging recalibration of histologic thresholds, particularly in settings where pathology remains the cornerstone of diagnosis in the absence of high-throughput molecular tools.

To mitigate potential overestimation, mitotic counts in our study—initially performed over 10 high-power fields (HPFs) using a 0.24 mm² field area—were standardized by converting to mitoses per mm², in line with recent cIMPACT-NOW recommendations (8) (61). This adjustment led to the downgrading of 10 cases: three from grade 2 to grade 1, and seven from grade 3 to grade 2. While such standardization is intended to improve grading reproducibility, it remains inconsistently applied across institutions and is not yet universally regulated. Whether these grading shifts reflect underlying biological behavior or merely technical variability remains uncertain and highlights the ongoing need for harmonized diagnostic criteria.

This study has several limitations. First, the cohort was heterogeneous, encompassing a broad range of age groups, anatomical locations (cranial convexity, skull base, spinal), and disease stages (both *de novo* and recurrent). Nonetheless, this heterogeneity was necessary to capture a comprehensive overview of the population’s characteristics. Second, although the commercial NGS panel provided broad oncologic coverage, it lacked certain meningioma-specific targets and did not assess large-scale chromosomal copy number alterations such as 1p and 22q loss, thereby limiting refined subclassification in some borderline tumors. Regarding clinical outcomes, the retrospective design and incomplete documentation prevented robust analysis of survival or progression-free intervals. This limitation was further compounded by structural challenges specific to our healthcare setting, including inconsistent follow-up, particularly among expatriate patients and patients who seek treatment abroad. Taken together, these constraints reflect the practical diagnostic realities faced by many institutions, particularly in settings where access to customized gene panels or high-throughput molecular tools remains limited.

## 5 Conclusion

Despite these limitations, our study presents a relatively large, well-characterized cohort and provides the first comprehensive molecular dataset on meningiomas from Kuwait. The findings are consistent with established genomic patterns while offering region-specific insights from an underrepresented population. Genotype and WHO histologic grade showed correlation with tumor location, while recurrence rates were more closely associated with grade and the extent of surgical resection, with no significant differences observed across molecular subtypes. Furthermore, our results highlight key limitations in conventional histopathologic assessment—particularly regarding mitotic thresholds and brain invasion criteria—underscoring the need for continued refinement of diagnostic and prognostic frameworks. We believe this work supports the broader implementation of molecular profiling in neuropathology and serves as a reference point for future investigations in similarly resource-limited contexts.

## Data Availability

The data presented in the study are deposited in the Figshare repository, at: https://figshare.com/articles/dataset/_b_Clinicopathological_and_Molecular_Data_of_131_Meningiomas_2021_2023_Kuwait_b_/29980738?file=57408235, under the DOI: 10.6084/m9.figshare.29980738.
