# Supplementary material for: Molecular and histopathological landscape of 131 meningiomas: a retrospective institutional study with insights from cIMPACT-NOW
Source: Front Oncol. 2025 Aug 29;15:1648953. doi: 10.3389/fonc.2025.1648953 (PMC12427037; doi:10.3389/fonc.2025.1648953)
Supplement: Supplementary file 1 [file Table1.docx]

**SUPPLEMENTARY TABLE 1:** Genes included in the Oncomine Comprehensive Assay panel (OCA v3)

| Hotspot genes | | | | Full-length genes | | | Copy number genes | | Gene fusions (inter- and intragenic) | | |
| --- | --- | --- | --- | --- | --- | --- | --- | --- | --- | --- | --- |
| AKT1 | ESR1 | KIT | PDGFRB | ARID1A | FBXW7 | PTEN | AKT1 | FGFR4 | AKT2 | FGFR2 | NUTM1 |
| AKT2 | EZH2 | KNSTRN | PIK3CB | ATM | MLH1 | RAD50 | AKT2 | FLT3 | ALK | FGFR3 | PDGFRA |
| AKT3 | FGFR1 | KRAS | PIK3CA | ATR | MRE11 | RAD51 | AKT3 | IGF1R | AR | FGR | PDGFRB |
| ALK | FGFR2 | MAGOH | PPP2R1A | ATRX | MSH6 | RAD51B | ALK | KIT | AXL | FLT3 | PIK3CA |
| AR | FGFR3 | MAP2K1 | PTPN11 | BAP1 | MSH2 | RAD51C | AXL | KRAS | BRCA1 | JAK2 | PRKACA |
| ARAF | FGFR4 | MAP2K2 | RAC1 | BRCA1 | NBN | RAD51D | AR | MDM2 | BRCA2 | KRAS | PRKACB |
| AXL | FLT3 | MAP2K4 | RAF1 | BRCA2 | NF1 | RNF43 | BRAF | MDM4 | BRAF | MDM4 | PTEN |
| BRAF | FOXL2 | MAPK1 | RET | CDK12 | NF2 | RB1 | CCND1 | MET | CDKN2A | MET | PPARG |
| BTK | GATA2 | MAX | RHEB | CDKN1B | NOTCH1 | SETD2 | CCND2 | MYC | EGFR | MYB | RAD51B |
| CBL | GNA11 | MDM4 | RHOA | CDKN2A | NOTCH2 | SLX4 | CCND3 | MYCL | ERBB2 | MYBL1 | RAF1 |
| CCND1 | GNAQ | MED12 | ROS1 | CDKN2B | NOTCH3 | SMARCA4 | CCNE1 | MYCN | ERBB4 | NF1 | RB1 |
| CDK4 | GNAS | MET | SF3B1 | CHEK1 | PALB2 | SMARCB1 | CDK2 | NTRK1 | ERG | NOTCH1 | RELA |
| CDK6 | H3F3A | MTOR | SMAD4 | CREBBP | PIK3R1 | STK11 | CDK4 | NTRK2 | ESR1 | NOTCH4 | RET |
| CHEK2 | HIST1H3B | MYC | SMO | FANCA | PMS2 | TP53 | CDK6 | NTRK3 | ETV1 | NRG1 | ROS1 |
| CSF1R | HNF1A | MYCN | SPOP | FANCD2 | POLE | TSC1 | EGFR | PDGFRA | ETV4 | NTRK1 | RSPO2 |
| CTNNB1 | HRAS | MYD88 | SRC | FANCI | PTCH1 | TSC2 | ERBB2 | PDGFRB | ETV5 | NTRK2 | RSPO3 |
| DDR2 | IDH1 | NFE2L2 | STAT3 |  |  |  | ESR1 | PIK3CB | FGFR1 | NTRK3 | TERT |
| EGFR | IDH2 | NRAS | TERT |  |  |  | FGF19 | PIK3CA |  |  |  |
| ERBB2 | JAK1 | NTRK1 | TOP1 |  |  |  | FGF3 | PPARG |  |  |  |
| ERBB3 | JAK2 | NTRK2 | U2AF1 |  |  |  | FGFR1 | RICTOR |  |  |  |
| ERBB4 | JAK3 | NTRK3 | XPO1 |  |  |  | FGFR2 | TERT |  |  |  |
| ERCC2 | KDR | PDGFRA |  |  |  |  | FGFR3 |  |  |  |  |
