# Supplementary material for: Molecular and histopathological landscape of 131 meningiomas: a retrospective institutional study with insights from cIMPACT-NOW
Source: Front Oncol. 2025 Aug 29;15:1648953. doi: 10.3389/fonc.2025.1648953 (PMC12427037; doi:10.3389/fonc.2025.1648953)
Supplement: Supplementary file 2 [file Table2.docx]

**SUPPLEMENTARY TABLE 2:** Annotated genetic variants and their allelic frequency in the tumor DNA samples (total = 128 samples)

| ID | Tumor location | Grade | Gene group | Gene | Coding | Amino acid | Variant type | Allele frequency | Oncogenicity | Variant ID | Gene amp/copy number | CDKN2A/B loss |
| --- | --- | --- | --- | --- | --- | --- | --- | --- | --- | --- | --- | --- |
| 6 | Spinal thoracic | 1 | NF2 | NF2 | c.1406delA | p.K469fs | frameshift del | 52.98% | Likely oncogenic | NA | _ | _ |
| 7 | Parietal convexity | 2 | NF2 | NF2 | c.691G>T | p.E231* | nonsense | 47.30% | Likely oncogenic | COSM26800 | ATM/5.83, MET/6.07 | NA |
| 8 | Temporal convexity | 3 | Non-NF2 | BRAF | c.1799T>A | p.V600E | missense | 62.37% | Oncogenic | COSM476 | _ | NA |
|  |  |  |  | BRCA1 | c.5137+1G>A | - | splice site | 48.05% | Likely oncogenic | NA |  |  |
| 9 | Cavernous sinus | 3 | NF2 | NF2 | c.634C>T | p.Q212* | nonsense | 89.94% | Likely oncogenic | COSM22240 | _ | NA |
| 10 | Spinal thoracic | 1 | NF2 | NF2 | c.784delC | p.R262fs | frameshift del | 47.02% | Likely oncogenic | NA | _ | _ |
| 11 | Multiple: temporal convexity, tentorium cerebelli | 3 | Non-NF2 | FBXW7 | c.1947G>A | p.W649* | nonsense | 91.70% | Likely oncogenic | COSM22933 | _ | NA |
|  |  |  |  | MED12 | c.131G>A | p.G44D | missense | 22.73% | Oncogenic | COSM131596 |  |  |
| 12 | Anterior clinoid | 2 | Non-NF2 | FANCI | c.2700delA | p.G901fs | frameshift del | 23.63% | Likely oncogenic | NA | _ | NA |
| 13 | Sphenoid wing | 2 | NF2 | NF2 | c.1127_1128insCG | p.S377fs | frameshift ins | 56.35% | Likely oncogenic | NA | _ | _ |
| 15 | Parietal convexity | 3 | NF2 | NF2 | c.1021C>T | p.R341* | nonsense | 52.90% | Likely oncogenic | COSM21990 | _ | NA |
| 16 | Parietal falcine/ parasagittal | 2 | Neg | _ | _ | _ | _ | _ | _ | _ | _ | _ |
| 17 | Multiple: frontal & parietal convexities, falcine | 2 | NF2 | NF2 | c.1228C>T | p.Q410* | nonsense | 47.95% | Likely oncogenic | COSM22209 | _ | _ |
| 18 | Frontal falcine/ parasagittal | 3 | Non-NF2 | Amplifica-tions | _ | _ | _ | _ | _ | _ | TERT/18.4, CCND3/16.1, MYC/12.4, IGF1R/11.4, RICTOR/10.8 | NA |
| 19 | Multiple: frontal convexity, parietal convexity, bilat cerebellar convexity | 2 | Neg | _ | _ | _ | _ | _ | _ | _ | _ | _ |
| 20 | Parietal convexity | 3 | NF2 | NF2 | c.600-3C>G | - | splice site | 63.81% | Likely oncogenic | NA | _ | _ |
| 21 | Frontal falcine/ parasagittal | 2 | NF2 | NF2 | c.169C>T | p.R57* | nonsense | 39.22% | Likely oncogenic | COSM1716332 | _ | _ |
| 23 | Parieto-temporal convexity | 2 | Neg | _ | _ | _ | _ | _ | _ | _ | _ | _ |
| 24 | Sphenoid wing | 2 | Neg | _ | _ | _ | _ | _ | _ | _ | _ | _ |
| 25 | Frontal convexity | 2 | NF2 | NF2 | c.600-1G>T​ | - | splice site | 43.21% | Likely oncogenic | NA | _ | _ |
| 26 | Tentorium cerebelli | 1 | Non-NF2 | AKT1 | c.49G>A | p.E17K | missense | 41.14% | Oncogenic | COSM33765 | _ | _ |
|  |  |  |  | NOTCH3 | c.5914-1G>AG | NA | splice acceptor | 52.72% | Likely oncogenic | NA |  |  |
| 27 | Parietal convexity | 2 | Non-NF2 | AKT1 | c.986C>G | p.A329G | missense | 42.60% | Oncogenic | NA | _ | _ |
| 28 | Fronto-parietal convexity | 2 | Neg | _ | _ | _ | _ | _ | _ | _ | _ | _ |
| 29 | Frontal falcine/ parasagittal | 2 | NF2 | NF2 | c.334G>T | p.E112* | nonsense | 28.10% | Likely oncogenic | COSM5611420 | _ | _ |
| 30 | Parietal convexity | 2 | NF2 | NF2, | c.744_745insC | p.R249fs | frameshift ins | 39.24% | Likely oncogenic | NA | _ | _ |
|  |  |  |  | TP53 | c.215_216insG | p.V73fs | frameshift ins | 100.00% | Likely oncogenic | COSM9311910 |  |  |
| 31 | Parietal falcine/ parasagittal | 2 | Non-NF2 | SMARCB1 | c.1130G>A | p.R377H | missense | 54.30% | Likely oncogenic | COSM989 | _ | _ |
|  |  |  |  | TP53 | c.215_216insG | p.V73fs | frameshift ins | 100.00% | Likely oncogenic | COSM9311910 |  |  |
| 32 | Anterior clinoid | 2 | Non-NF2 | SMO | c.1234C>T | p.L412F | missense | 41.99% | Oncogenic | COSM216037 | _ | _ |
| 33 | Planum sphenoidale | 1 | Non-NF2 | AKT1 | c.49G>A | p.E17K | missense | 22.97% | Oncogenic | COSM33765 | _ | _ |
| 34 | Olfactory groove | 2 | Non-NF2 | SMO | c.1234C>T | p.L412F | missense | 39.50% | Oncogenic | COSM216037 | _ | _ |
| 35 | Olfactory groove | 2 | Neg | _ | _ | _ | _ | _ | _ | _ | _ | _ |
| 36 | Frontal convexity | 2 | Neg | _ | _ | _ | _ | _ | _ | _ | _ | _ |
| 37 | Sphenoid wing | 1 | Non-NF2 | TRAF7 | c.1921C>T | p.R641C | missense | 34.43% | Oncogenic | COSM1578635 | _ | _ |
|  |  |  |  | KLF4 | c.1225A>C | p.K409Q | missense | 46.00% | Likely oncogenic | COSM248828 |  |  |
| 39 | Olfactory groove | 2 | Non-NF2 | BRCA2 | c.7534delC | p.A2513fs | frameshift del | 42.56% | Likely oncogenic | ClinVar 1696809 | _ | _ |
| 40 | Cerebellopontine angle | 2 | Non-NF2 | TP53 | c.215_216insG | p.V73fs | frameshift ins | 100.00% | Likely oncogenic | COSM9311910 | _ | _ |
| 41 | Sphenoid wing | 2 | Non-NF2 | AKT1 | c.49G>A | p.E17K | missense | 32.59% | Oncogenic | COSM33765 | _ | _ |
| 42 | Olfactory groove | 1 | Non-NF2 | TRAF7 | c.1961G>A | p.G654D | missense | 29.50% | Likely oncogenic | NA | _ | _ |
|  |  |  |  | PMS2 | c.2192_2196del | p.L731fs | frameshift del | NA | Oncogenic | RCV000076846 |  |  |
| 43 | Frontal convexity | 1 | Non-NF2 | PIK3CA | c.3140A>G | p.H1047R | missense | 30.25% | Oncogenic | COSM775 | _ | _ |
| 44 | Frontal falcine/ parasagittal | 1 | NF2 | NF2 | c.41_42delTC | p.L14fs | frameshift del | 30.07% | Likely oncogenic | COSM22312 | _ | _ |
| 45 | Frontal convexity | 2 | NF2 | NF2 | c.732delT | p.Y244* | nonsense | 51.81% | Likely oncogenic | COSM23866 | _ | _ |
| 46 | Planum sphenoidale | 1 | Neg | _ | _ | _ | _ | _ | _ | _ | _ | _ |
| 47 | Frontal convexity | 2 | Neg | _ | _ | _ | _ | _ | _ | _ | _ | _ |
| 48 | Planum sphenoidale | 1 | Neg | _ | _ | _ | _ | _ | _ | _ | _ | _ |
| 49 | Parietal convexity | 2 | Non-NF2 | SMARCB1 | c.1130G>A | p.R377H | missense | 43.07% | Likely oncogenic | COSM989 | _ | _ |
| 50 | Olfactory groove | 1 | Neg | _ | _ | _ | _ | _ | _ | _ | _ | _ |
| 51 | Sphenoid wing | 1 | Neg | _ | _ | _ | _ | _ | _ | _ | _ | _ |
| 52 | Sphenoid wing | 1 | NF2 | NF2 | c.114+2T>C | - | splice donor | 42.08% | Likely oncogenic | COSM9271437 | _ | _ |
| 53 | Sphenoid wing | 1 | Non-NF2 | TRAF7 | c.1570C>T | p.R524W | missense | 39.95% | Oncogenic | COSM6807293 | _ | _ |
|  |  |  |  | KLF4 | c.1225A>C | p.K409Q | missense | 41.30% | Likely oncogenic | COSM248828 |  |  |
| 54 | Sphenoid wing | 2 | Non-NF2 | AKT1 | c.49G>A | p.E17K | missense | 34.09% | Oncogenic | COSM33765 | _ | _ |
| 55 | Fronto-parietal convexity | 2 | NF2 | NF2 | c.1396C>T | p.R466* | nonsense | 48.06% | Likely oncogenic | COSM23667 | _ | Heterozygous del |
| 56 | Frontal convexity | 2 | Neg | _ | _ | _ | _ | _ | _ | _ | _ | _ |
| 57 | Frontal convexity | 2 | Non-NF2 | TRAF7 | c.1958G>A | p.R653Q | missense | 34.10% | Likely oncogenic | COSM1578113 | _ | _ |
|  |  |  |  | KLF4 | c.1225A>C | p.K409Q | missense | 33.70% | Likely oncogenic | COSM248828 |  |  |
| 58 | Petroclival | 1 | Non-NF2 | AKT1 | c.49G>A | p.E17K | missense | 20.89% | Oncogenic | COSM33765 | _ | _ |
| 59 | Tuberculum sellae | 1 | Non-NF2 | AKT1 | c.49G>A | p.E17K | missense | 36.39% | Oncogenic | COSM33765 | _ | _ |
| 60 | Frontal falcine/ parasagittal | 2 | NF2 | NF2 | c.193C>T | p.Q65* | nonsense | 32.56% | Likely oncogenic | COSM22328 | _ | _ |
| 61 | Sphenoid wing | 2 | Non-NF2 | PIK3CA | c.1633G>A | p.E545K | missense | 49.52% | Oncogenic | COSM763 | _ | NA |
| 62 | Anterior clinoid | 1 | Neg | _ | _ | _ | _ | _ | _ | _ | _ | _ |
| 63 | Temporal convexity | 2 | NF2 | NF2 | c.122G>A | p.W41* | nonsense | 74.30% | Likely oncogenic | COSM144189 | _ | Heterozygous del |
| 64 | Frontal convexity | 2 | Neg | _ | _ | _ | _ | _ | _ | _ | _ | _ |
| 65 | Frontal falcine/ parasagittal | 2 | Neg | _ | _ | _ | _ | _ | _ | _ | _ | _ |
| 66 | Frontal convexity | 1 | Non-NF2 | AKT1 | c.49G>A | p.E17K | missense | 34.20% | Oncogenic | COSM33765 | _ | _ |
|  |  |  |  | SMO | c.596G>A | p.R199Q | missense | 47.12% | Unknown | COSM6493918 |  |  |
| 67 | Frontal falcine/ parasagittal | 2 | NF2 | NF2 | c.1247delC | p.A416fs | frameshift del | 31.13% | Likely oncogenic | NA | _ | _ |
| 68 | Parietal convexity | 3 | NF2 | NF2 | c.447+3A>C | - | splice site | 59.73% | Likely oncogenic | NA | _ | Homozygous del |
| 69 | Sphenoid wing | 2 | NF2 | NF2 | c.71_72insT | p.R25fs | frameshift ins | 52.70% | Likely oncogenic | COSM22281 | _ | _ |
| 70 | Two: olfactory groove, frontal falcine | 2 | Non-NF2 | AKT1 | c.49G>A | p.E17K | missense | 35.89% | Oncogenic | COSM33765 | _ | _ |
| 71 | Olfactory groove | 2 | Neg | _ | _ | _ | _ | _ | _ | _ | _ | _ |
| 72 | Two: frontal convexity, frontal falcine | 2 | NF2 | NF2 | c.1300G>T | p.E434* | nonsense | 52.13% | Likely oncogenic | COSM6848125 | _ | _ |
| 73 | Parietal convexity | 1 | NF2 | NF2 | c.346_348del | p.H116del | nonframeshift del | 37.80% | VUS | NA | _ | _ |
| 74 | Spinal lumbar | 2 | Neg | _ | _ | _ | _ | _ | _ | _ | _ | _ |
| 75 | Frontal convexity | 2 | NF2 | NF2 | c.448-1G>A | - | splice acceptor | 73.06% | Likely oncogenic | COSM24209 | _ | _ |
| 76 | Frontal convexity | 2 | Non-NF2 | STK11 | c.369delG | p.M125fs | frameshift del | 50.65% | Likely oncogenic | NA | _ | _ |
| 77 | Spinal thoracic | 1 | NF2 | NF2 | c.784C>T | p.R262* | nonsense | 44.60% | Likely oncogenic | COSM22000 | _ | _ |
| 78 | Frontal falcine/ parasagittal | 1 | Neg | _ | _ | _ | _ | _ | _ | _ | _ | _ |
| 79 | Multiple: spinal, frontal falcine & convexity, parietal falcine & convexity, CPA, tentorium | 2 | NF2 | NF2 | c.599+1G>A | - | splice donor | 44.18% | Likely oncogenic | COSM24550 | _ | _ |
| 80 | CPA | 1 | Non-NF2 | PIK3CA | c.3140A>G | p.H1047R | missense | 39.80% | Oncogenic | COSM775 | _ | _ |
| 81 | Occipital convexity | 2 | NF2 | NF2 | c.347_348insA | p.H116fs | frameshift ins | 70.06% | Likely oncogenic | NA | _ | _ |
| 82 | Frontal convexity | 2 | Non-NF2 | ATM | c.3077+1G>C | - | splice donor | 54.25% | Likely oncogenic | COSM1756728 | _ | _ |
| 83 | Planum sphenoidale | 1 | Non-NF2 | TRAF7 | c.1606G>A | p.G536S | missense | 42.30% | Likely oncogenic | COSM1578118 | _ | _ |
| 84 | Cavernous sinus | 2 | Neg | _ | _ | _ | _ | _ | _ | _ | _ | _ |
| 85 | Two: cavernous sinus, spinal cervical | 1 | Non-NF2 | AR | c.2146G>A | p.V716M | missense | 34.55% | Likely oncogenic | COSM3094417 | _ | _ |
| 86 | Parietal convexity | 2 | NF2 | NF2 | c.1282delC | p.Q428fs | frameshift del | 49.60% | Likely oncogenic | NA | _ | _ |
| 87 | Parietal falcine/ parasagittal | 2 | NF2 | NF2 | c.915_925del | p.D305fs | frameshift del | 51.67% | Likely oncogenic | COSM22434 | _ | _ |
| 88 | Olfactory groove | 2 | Non-NF2 | AKT1 | c.49G>A | p.E17K | missense | 30.31% | Oncogenic | COSM33765 | _ | _ |
| 89 | Sphenoid wing | 2 | Neg | _ | _ | _ | _ | _ | _ | _ | _ | Heterozygous del |
| 90 | Frontal convexity | 2 | NF2 | NF2 | c.593delG | p.R198fs | frameshift del | 34.27% | Likely oncogenic | NA | _ | _ |
| 91 | Frontal convexity | 1 | Neg | _ | _ | _ | _ | _ | _ | _ | _ | _ |
| 92 | Sphenoid wing | 1 | Neg | _ | _ | _ | _ | _ | _ | _ | _ | _ |
| 93 | Parietal convexity | 2 | NF2 | NF2 | c.1357_1358delCA | p.Q453Afs*41 | frameshift del | 47.71% | Likely oncogenic | NA | _ | _ |
| 94 | Spinal thoracic | 1 | Non-NF2 | SMARCB1 | c.1130G>A | p.R377H | missense | 11.31% | Likely oncogenic | COSM989 | _ | _ |
| 95 | Planum sphenoidale | 1 | Non-NF2 | TRAF7 | c.1198T>A | p.W400R | missense | 31.57% | Likely oncogenic | COSM6985402 | _ | _ |
| 96 | Frontal convexity | 2 | NF2 | NF2 | c.1219delC | p.Q407fs | frameshift del | 52.29% | Likely oncogenic | NA | _ | _ |
| 97 | Multiple: sphenoid wing, convexity, falcine, posterior fossa | 2 | NF2 | NF2 | Heterozygous 22q12.2 loss | Heterozy-gous 22q12.2 loss | Structural variant, loss of exon 1-16 | NA | Oncogenic | NA | _ | _ |
| 98 | Parietal convexity | 1 | NF2 | NF2 | c.815delC | p.T272fs | frameshift del | 44.58% | Likely oncogenic | COSM1685316 | _ | _ |
| 99 | Multiple: ant clinoid, olfactory groove, frontal falcine | 2 | Neg | _ | _ | _ | _ | _ | _ | _ | _ | _ |
| 100 | Anterior clinoid | 1 | Neg | _ | _ | _ | _ | _ | _ | _ | _ | _ |
| 101 | Olfactory groove | 2 | Non-NF2 | PIK3CA | c.1633G>A | p.E545K | missense | 12.20% | Oncogenic | COSM763 | _ | _ |
|  |  |  |  | ARID1A | c.3154_3155insT | p.Y1052fs | frameshift ins | 8.33% | Likely oncogenic | NA |  |  |
| 102 | Spinal thoracic | 1 | NF2 | NF2 | c.1022delG | p.R341fs | frameshift del | 50.38% | Likely oncogenic | NA | _ | _ |
| 103 | Sphenoid wing | 3 | NF2 | NF2 | c.1042_1043insG | p.E348fs | frameshift ins | 64.98% | Likely oncogenic | NA | _ | _ |
| 104 | Frontal falcine/ parasagittal | 2 | NF2 | NF2 | c.947delT | p.L316fs | frameshift del | 54.70% | Likely oncogenic | COSM9271319 | _ | _ |
|  |  |  |  | TP53 | c.736A>G | p.M246V | missense | 36.80% | Likely oncogenic | COSM43555 |  |  |
| 105 | Anterior clinoid | 1 | Neg | _ | _ | _ | _ | _ | _ | _ | _ | _ |
| 106 | Sphenoid wing | 1 | NF2 | NF2 | c.432_433insAC | p.A145fs | frameshift ins | 60.79% | Likely oncogenic | NA | _ | _ |
| 107 | Fronto-parietal convexity | 2 | NF2 | NF2 | c.1357delC | p.Q453fs | frameshift del | 52.39% | Likely oncogenic | COSM23746 | _ | _ |
| 108 | Sphenoid wing | 1 | Neg | _ | _ | _ | _ | _ | _ | _ | _ | _ |
| 109 | Olfactory groove | 2 | Neg | _ | _ | _ | _ | _ | _ | _ | _ | _ |
| 110 | Petroclival | 1 | Neg | _ | _ | _ | _ | _ | _ | _ | _ | _ |
| 111 | Frontal convexity | 2 | Non-NF2 | Amplifica-tion | _ | _ | _ | _ | _ | _ | SMARCA4/7.59 | _ |
| 112 | Sphenoid wing | 2 | Non-NF2 | SMARCB1 | c.1121G>A | p.R374Q | missense | 62.48% | Oncogenic | COSM998 | _ | _ |
| 113 | Frontal falcine/ parasagittal | 2 | Non-NF2 | AKT1 | c.49G>A | p.E17K | missense | 31.86% | Oncogenic | COSM33765 | _ | _ |
| 114 | Parietal convexity | 2 | Non-NF2 | AKT1 | c.49G>A | p.E17K | missense | 32.15% | Oncogenic | COSM33765 | _ | _ |
| 115 | Fronto-parietal convexity | 2 | Neg | _ | _ | _ | _ | _ | _ | _ | _ | _ |
| 116 | Frontal convexity | 3 | Neg | _ | _ | _ | _ | _ | _ | _ | _ | _ |
| 117 | Frontal convexity | 2 | NF2 | NF2 | c.1446+1G>T | - | splice donor | 58.70% | Likely oncogenic | NA | _ | _ |
| 118 | Two: planum sphenoidale, ant clinoid | 1 | Neg | _ | _ | _ | _ | _ | _ | _ | _ | _ |
| 119 | Sphenoid wing | 2 | Non-NF2 | PTEN | c.1003C>T | p.R335* | nonsense | 43.99% | Oncogenic | COSM5151 | _ | _ |
|  |  |  |  | FANCA | c.3239+1G>A | - | splice donor | 55.00% | Likely oncogenic | NA |  |  |
| 120 | Planum sphenoidale | 1 | Neg | _ | _ | _ | _ | _ | _ | _ | _ | _ |
| 121 | Tuberculum sellae | 1 | NF2 | NF2 | c.215T>C | p.V72A | missense | 49.80% | Likely oncogenic | NF2 ClinVar 569354 | _ | _ |
|  |  |  |  | PIKC3A | c.3131A>G | p.N1044S | missense | 31.33% | Likely oncogenic | COSM22541 |  |  |
| 122 | Olfactory groove | 1 | Neg | _ | _ | _ | _ | _ | _ | _ | _ | _ |
| 123 | Anterior clinoid | 1 | Non-NF2 | BRCA2 | c.67+2T>C | - | splice donor | 49.80% | Oncogenic | ClinVar 52163 | _ | _ |
| 124 | Frontal falcine/ parasagittal | 2 | NF2 | NF2 | c.1009C>T | p.Q337* | nonsense | 68.54% | Likely oncogenic | COSM22249 | _ | _ |
|  |  |  |  | SMARCB1 | c.1130G>A | p.R377H | missense | 67.14% | Likely oncogenic | COSM989 |  |  |
|  |  |  |  | STK11 | c.369delG | p.M125fs | frameshift del | 50.95% | Likely oncogenic | COSM10287028 |  |  |
| 125 | Sphenoid wing | 1 | Non-NF2 | AKT1 | c.49G>A | p.E17K | missense | 35.26% | Oncogenic | COSM33765 | _ | _ |
| 126 | Sphenoid wing | 1 | Non-NF2 | ATM | c.381delA | p.V128* | nonsense | 50.18% | Likely oncogenic | CliVar 141546 | _ | _ |
| 127 | Frontal convexity | 2 | Non-NF2 | ARID1A | c.5041delC | p.L1681fs | frameshift del | 28.15% | Likely oncogenic | NA | _ | _ |
| 128 | Occipital convexity | 1 | NF2 | NF2 | c.599+1G>T | - | splice donor | 32.99% | Likely oncogenic | COSM9271414 | _ | _ |
| 129 | Parieto-occipital convexity | 2 | Neg | _ | _ | _ | _ | _ | _ | _ | _ | _ |
| 130 | Spinal thoracic | 1 | NF2 | NF2 | c.784C>T | p.R262* | nonsense | 71.73% | Likely oncogenic | COSM22000 | _ | _ |
| 131 | Fronto-parietal convexity | 2 | Neg | _ | _ | _ | _ | _ | _ | _ | _ | _ |
| 132 | Frontal convexity | 2 | NF2 | NF2 | c.1375C>T | p.Q459* | nonsense | 46.10% | Likely oncogenic | COSM23884 | _ | _ |
| 133 | Sphenoid wing | 2 | Neg | _ | _ | _ | _ | _ | _ | _ | _ | _ |
| 134 | Olfactory groove | 2 | Non-NF2 | AKT1 | c.155T>G | p.L52R | missense | 30.56% | Oncogenic | COSM93893 | _ | _ |
| 135 | Fronto-temporal convexity | 1 | Non-NF2 | TP53 | c.902_903delCA | p.P301fs | frameshift del | 38.48% | Likely oncogenic | NA | _ | _ |
| 136 | CPA | 2 | Neg | _ | _ | _ | _ | _ | _ | _ | _ | _ |
